# Tension Strength of Multi-Fastener, Single-Lap Joints in Flax and Jute Composite Plates Using Bolts or Rivets

**DOI:** 10.3390/ma18102180

**Published:** 2025-05-08

**Authors:** Mike R. Bambach

**Affiliations:** Department of Civil Engineering, The University of Sydney, Sydney, NSW 2006, Australia; mike.bambach@sydney.edu.au

**Keywords:** jute, flax, fiber–epoxy composites, bolt connections, rivet connections, joints

## Abstract

The behavior of joints and fasteners in fiber-epoxy composites has been researched for several decades, and many studies have demonstrated their performance in tension testing. These studies have focused nearly exclusively on synthetic fibers, such as carbon and glass. Meanwhile, natural fiber–epoxy composites have recently received considerable attention as load-bearing members, including as columns and beams. In order for individual members to be used to create structural systems, the behavior of mechanically fastened joints in natural fiber–epoxy composites needs to be thoroughly investigated. This paper presents an experimental program of 120 single-lap joints in flax–epoxy and jute–epoxy composites. Between one and three mechanical fasteners were used in the joints, and both bolts and rivets were investigated. A variety of geometric variables were investigated, relevant to joints between load-bearing members. The results are used to demonstrate the optimum strength of multi-fastener joints in natural fiber composite structural systems. It is shown that maximum joint efficiency is achieved with larger fastener-diameter-to-width ratios, three fasteners (located along the line of action of the force), and edge-distance-to-fastener-diameter ratios greater than 2.5.

## 1. Introduction

Connections between load-carrying members form an important part of a structural system. Synthetic fiber–epoxy composites have been used in a wide variety of structural applications in various industries, such as aeronautical, automotive, marine, construction, wind energy, and sports industries. The topic of mechanical fasteners for structural joints in synthetic composites such as carbon and glass has been studied by many authors over several decades. Indeed, some research on the topic dates back to the 1970s, for example, [1,2]. In comparison with isotropic materials such as metals, orthotropic composites have unique material characteristics that result in a wide range of failure modes, such as bearing, matrix failure, hole elongation, tear-out, shear-out, local fiber–matrix splitting, delamination, net section fracture, tilting, splaying, and bending. It has been noted [3] that the ductility of metals relieves stress concentrations at the hole, while the lack of ductility in composites results in higher stresses at the hole than in isotropic materials. The joint efficiency has been identified as an important parameter to assess mechanically fastened joints, being the ratio of the joint strength to the Open-Hole Tension Strength (OHT). The OHT forms an upper-bound strength for mechanically fastened joints in composite structures [4,5]. Due to the confluence of the various failure mechanisms, single-fastener joints have been shown to have relatively low efficiencies. Gamdani [4] noted efficiencies of 31% to 54% for 36 mm wide samples of CFRP and GFRP, and 40% to 68% for 22 mm wide samples. Similarly low efficiencies for single-bolted joints were demonstrated further in Gamdani [5]. For this reason, many joint studies were extended to multi-bolted joints [6,7,8]. Several authors have demonstrated that many of the complex mechanisms associated with single-bolt joints are somewhat alleviated in multi-fastener joints, and multi-fastener joints have been shown to have strengths that may reach the (OHT), provided sufficient bolts are used [4,5].

**Figure 1:**
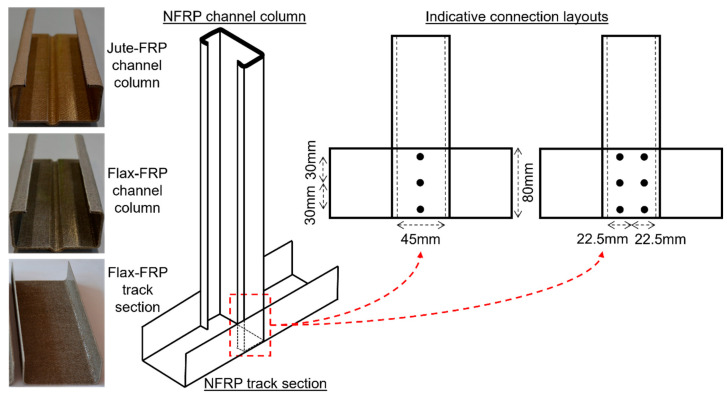
Exemplar connection between a channel section stud column and a channel section track member fabricated from natural fiber FRP (NFRP), consisting of either jute or flax fibers. Photos from previous studies of NFRP channel columns and tracks are from [22,23,24]. Connection layouts demonstrate indicative fastener locations, with either one row or two rows of fasteners located within the flat width of the channel column flange (45 mm).

More recently, natural fiber-reinforced composites have received considerable attention, particularly due to their favorable sustainability properties, such as low energy production, renewability, recyclability, biodegradability, and less hazardous manufacturing [9,10,11,12,13,14,15,16,17,18,19,20,21]. Natural fiber–epoxy composites such as those fabricated from flax and jute have been shown to have great potential in various non-structural and light structural applications, including in the automotive and construction industries [22,23,24,25,26,27,28,29,30,31,32,33]. A series of recent studies by the author have quantified the structural properties of flax–fiber and jute–fiber epoxy composites and demonstrated their applicability as structural members in residential and light commercial or industrial applications [22,23,24]. Notably, thin-walled channel sections were fabricated from flax and jute composites (see photos in Figure 1) and tested in either compression or bending, and were shown to match or exceed the structural properties of thin-walled steel members. These studies identified flax and jute composite channels as potential substitutes for steel framing in building applications that use light-gauge steel members.

In order to construct a structural system from flax and jute composite channel members, a detailed understanding of the behavior of mechanically fastened joints in such composites is required. There have been few studies directed towards the behavior of natural fiber composite joints [34,35,36,37]. These studies have demonstrated that several variables are important to the strength of bolted natural fiber composites, such as edge distance, diameter-to-width ratio, number of fasteners, bolt torque [34], and washer size [35]. Kaushik [36] identified non-conventional joining techniques in flax/PP laminated composites that outperformed bolts, including hybrid bolted–ultrasonic and hybrid bolted–hot plate joints. However, no studies have comprehensively studied mechanically fastened joints in flax and jute composites, relevant to the application of joints in structural framing systems.

It is the aim of the present paper to fill this knowledge gap and provide structural design guidance to optimize the joints. A broad experimental program was devised to understand the important parameters in such joints, including the effects of edge distance, fastener diameter, effective width of the composite (the width of composite that carries the load), number of fasteners, and type of fasteners. A total of 120 experiments were conducted, and the corresponding joint efficiencies were calculated and compared, with the goal of identifying joint parameters that create the most efficient joint.

## 2. Materials and Methods

### 2.1. Design of Single-Lap Joints for the Experimental Program

The previous studies of load-bearing natural fiber composite structural members [22,23,24] focused principally on channel-shaped sections that could potentially be used in light structural applications such as residential and light commercial constructions (see photos in Figure 1). One particular application is stud frames (load-bearing wall frame), for which a typical detail of the stud column to bottom track section is shown in Figure 1. Such connections are designed to resist uplift loads (for the case where the wind uplift force exceeds the self-weight gravity load), and bending loads due to the lateral wind pressure.

**Figure 2:**
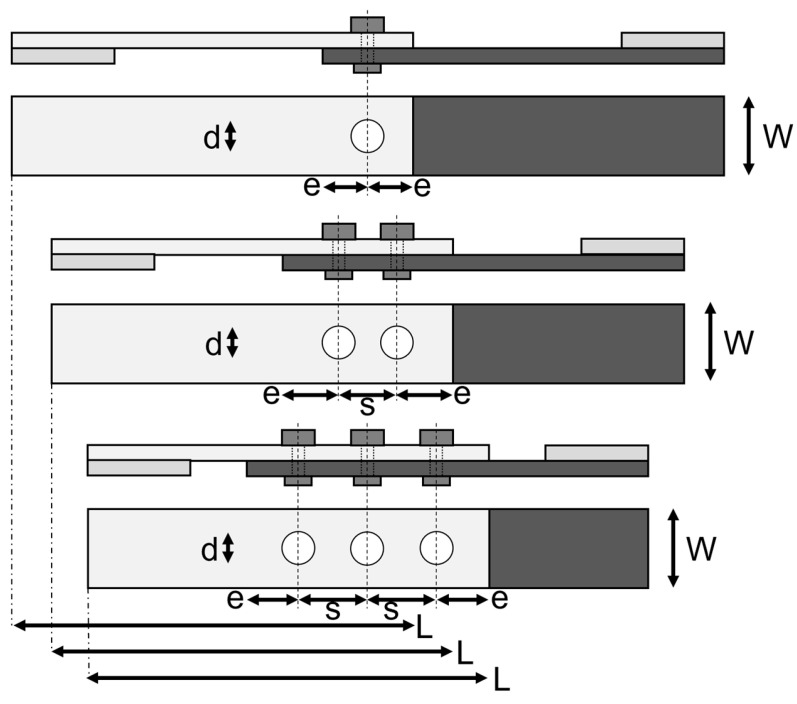
Experimental joints with 1, 2, or 3 fasteners along the line of action of the force.

Figure 1 shows two indicative connection designs, with either 1 or 2 rows of fasteners parallel to the line of action of the force. The figure shows 3 fasteners in each row; however, such a joint could potentially consist of 1, 2, or 3 fasteners in a row depending on the design capacity required. The flange of the channel column is connected to the flange of the track section, such that a single-lap joint is considered appropriate. Accordingly, the 3 basic joint configurations selected for the experimental program are those shown in Figure 2.

In residential and light commercial framing, several types of fasteners are typically used, depending on whether the members are timber or light-gauge steel, nails, screws, bolts, and rivets. In the present study, where the joined materials are relatively thin (3.5 mm), bolts and rivets were considered. Self-tapping screws are outside the scope of this study since it is not clear how such screws will interact with the fiber–resin composite materials. For the design of such joints in light-gauge steel, the edge distance (*e*) is an important parameter, since fasteners located close to an edge can precipitate edge tear-out failures which are not efficient. In accordance with Figure 1, the effective width of the joint (representing the width of flange that carries the bolt load) is either 45 mm or 22.5 mm, and in the present study, 2 values of width (*W*) of 45 mm (wide) and 25 mm (narrow) were considered. The majority of fastener diameters considered in this study were 6 mm for bolts and 6.4 mm for rivets; however, a small number of 4 mm bolts were also used for comparison purposes. The fastener spacing (*s*) was taken as 30 mm in all cases. The joints with 1 and 2 fasteners had sample lengths (*L*) of 150 mm, while the 3 fastener joints were 200 mm. The clearance holes for the 6 mm and 6.4 mm fasteners were 7 mm, and for the 4 mm bolts, they were 5 mm. The clearance holes were drilled with a low-speed drill with a hardened steel drill bit designed for drilling into steel. The specific combinations of variables considered are shown in Table A1 and Table A2 in Appendix A, where 60 tests were undertaken for the wide samples (*W* = 45 mm), and 60 for the narrow samples (*W* = 25 mm).

It is noted that there is an ASTM specification for single-lap tension testing of fastened joints in composite laminates (ASTM D5961 [38]); however, in the present study, the geometries considered important were those relative to the application demonstrated in Figure 1, and the ASTM method was not considered appropriate for this scope.

### 2.2. Materials

The natural fiber–epoxy composite materials used in the present study were fabricated similarly to the previous experiments on plates and channel sections by the author [22,23,24]. The fibers were commercially available flax and jute woven fabrics from Composites Evolution (Chesterfield, UK) (Biotex Flax and Biotex Jute), with an arial density of 400 g/m^2^ and a 2 × 2 twill weave. Nominal density, tensile strength, and modulus values for the flax were 1.5 g/cm^3^, 500 MPa, and 50 GPa, and for the jute, they were 1.46 g/cm^3^, 400 MPa, and 40 GPa. The epoxy resin was a commercially available bulk laminating resin Kinetix R240 (ATL Composites, Molendinar, Australia), with a fast hardener. The samples were fabricated with a traditional hand-layup process, wherein 4 layers of fabric were wet-out by hand and then placed under a vacuum. The fabricated plates were 1200 × 300 mm, with a mean nominal thickness of 3.5 mm, from which the samples were cut using a water-jet cutter, with dimensions of *W* × *L*.

Following fabrication of the flax and jute composites, the fiber volume fraction was estimated from the mass of fabric prior to fabrication, the mass of composite after fabrication, and the constituent densities. The average fiber volume fraction of the flax composites was 45.3%. The average fiber volume fraction of the jute composites was 42.8%.

The material properties of the fabricated flax and jute composites were established from tension tests undertaken in accordance with ISO 527-4 [39]. In accordance with this procedure, a sample width of 25 mm was used. Samples of 25 mm width were then prepared with a centrally located hole of diameter 7 mm and tested in pure tension. These tests were undertaken by clamping the two ends of the sample in the machine grips and loading the sample in pure tension, at a rate of 1 mm/min.

The 6 mm bolts were steel Grade 8.8 bolts with washers of outside diameter 17 mm. The 4 mm bolts were steel Grade 8.8 bolts with washers of outside diameter 12 mm, and they were installed into holes of diameter 5 mm. The 6.4 mm blind rivets had heads of outside diameter 12 mm. The bolts were installed finger-tight. The finger-tight installation ensures that the clamping force is negligible, such that the transfer of load is one of pure bolt shear rather than bolt shear and friction. The blind rivets were installed with a standard rivet tool, which generates a compression force between the joined plates due to the compression between the rivet head and the mandrel head, resulting from the expansion of the blind end of the rivet during insertion. In all cases, the fasteners were considerably stronger than the composites they joined, such that no failures nor deformations were observed in any fasteners during the 120 tests. It was shown in [37] that conventional mechanical drilling methods are still used as primary applications for natural fiber composites, and they are the first choice among researchers.

### 2.3. Experimental Setup

**Figure 3:**
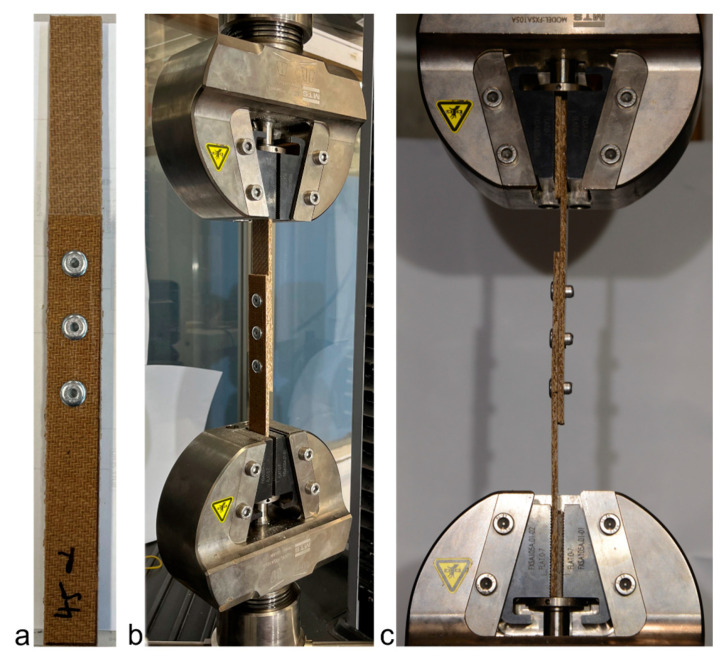
Experimental setup: (**a**) narrow jute composite joint with 3 rivets, (**b**) sample in the testing machine, and (**c**) side view of the test.

The experiments were undertaken on a 50 kN MTS Criterion testing machine (MTS Systems, Eden Prairie, MN, USA). A jute composite sample with 3 rivets is shown in Figure 3a, and setup in the testing machine is shown in Figure 3b,c. Within each upper and lower grip, a packing plate of the same width as the sample was used to ensure the sample was straight, as shown in Figure 2 and Figure 3c. The tests were undertaken in pure tension at a rate of 1 mm/min. Force and displacement acquisition was undertaken at a rate of 10 Hz.

## 3. Results

### 3.1. Material Properties of the Flax and Jute Composites

The mean nominal thickness for all flax and jute composite plates was 3.5 mm. The average tension ultimate stress of samples without a hole was 73.1 MPa for flax and 69.1 MPa for jute, and is referred to as the Tension Strength (TS). For samples with a hole, the ultimate stress was calculated according to Equation (1) and is referred to as the Open-Hole Tension (OHT) strength. In Equation (1), the ultimate load in the test is denoted as *P_ult_*, and *A_reduced_* is the reduced cross-section area calculated through the section with the hole. The average tension ultimate stress of the samples with a 7 mm hole was 62.4 MPa for flax and 51.9 MPa for jute. The ratio of the Open-Hole Tension Strength to the standard Tension Strength (OHT/TS) is an important indicator of the notch sensitivity of composite materials [4,5]. The ratio values were 0.86 for flax and 0.75 for jute.

*OHT = P_ult_/A_reduced_* (1)

**Figure 4:**
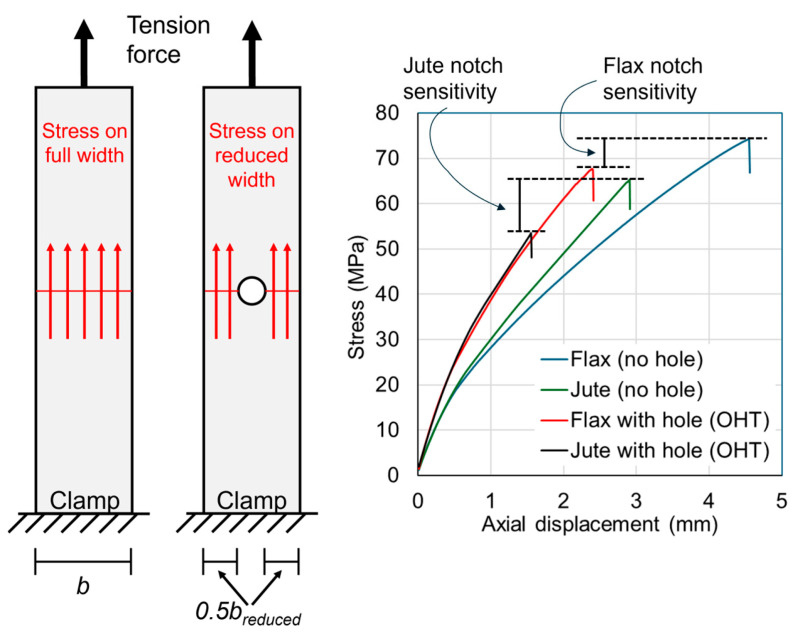
Comparisons between samples with no holes and samples with one 7 mm hole. The plot of stress versus axial displacement demonstrates the difference in strength due to the notch sensitivity of the composites.

The 25 mm wide samples with no hole are compared with the 25 mm wide samples with a 7 mm diameter hole in Figure 4. The plots of the samples with no hole indicate that the flax and jute composites are not linear elastic, and this is also evident with the samples with a hole. The notch sensitivity is evident in the plot as the reduction in the ultimate stress resulting from the addition of the hole. The diagram in Figure 4 demonstrates the different longitudinal stress state between these two samples, wherein the stress acts on the reduced width in the sample with a hole. It is also noted that the plot in Figure 4 illustrates the different ductility levels of the flax and jute, where the flax is more ductile.

### 3.2. Efficiency of the Fastened Joints

**Figure 5:**
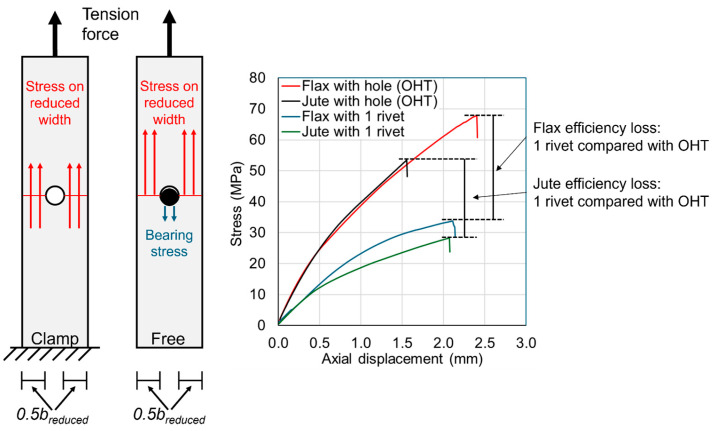
Comparisons between clamped samples with one hole and joints with one rivet. The plot of stress versus axial displacement demonstrates the difference in strength of the composites due to the fastener (i.e., the joint efficiency).

**Figure 6:**
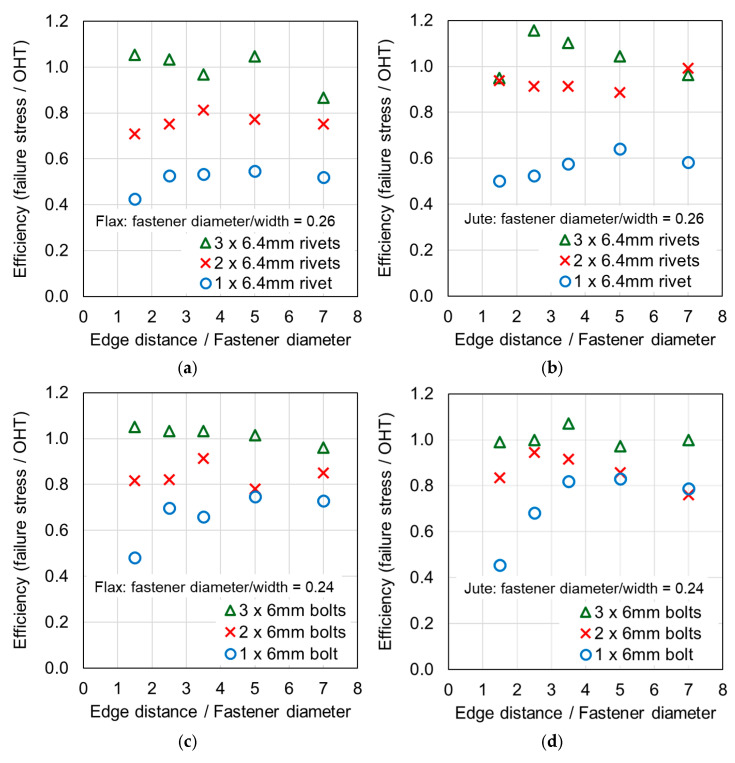
Plots of the composite efficiency versus ratio of the edge distance to the fastener diameter for the narrow samples: (**a**) riveted joints in flax composite, (**b**) riveted joints in jute composite, (**c**) bolted joints in flax composite, and (**d**) bolted joints in jute composite.

**Figure 7:**
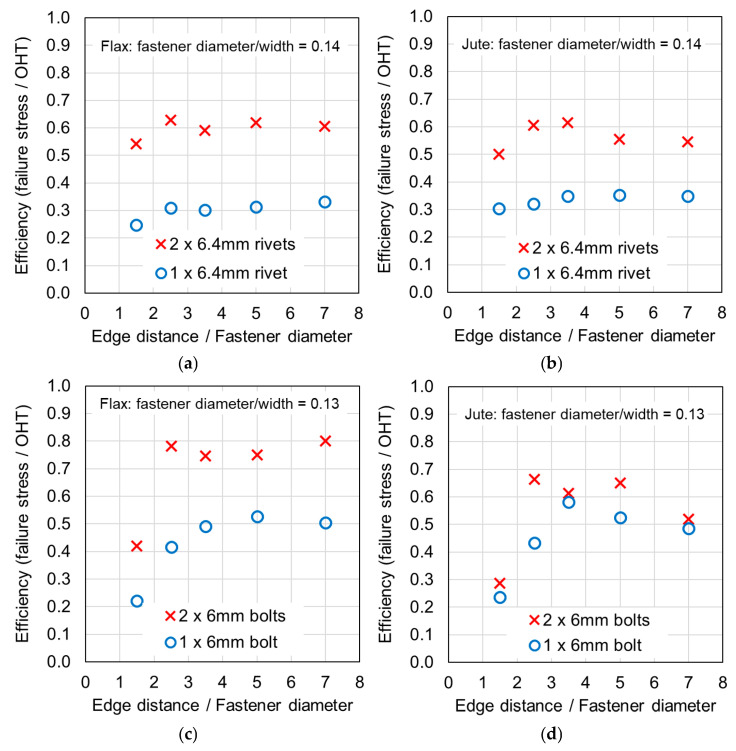
Plots of the composite efficiency versus ratio of the edge distance to the fastener diameter for the wide samples: (**a**) riveted joints in flax composite, (**b**) riveted joints in jute composite, (**c**) bolted joints in flax composite, and (**d**) bolted joints in jute composite.

Another important indicator for joints in composite materials using fasteners is the efficiency of the fastened joint. The efficiency is the ratio of the single-lap fastened joint strength (FJ) to the Open-Hole Tension Strength, where FJ is given in Equation (2). In Equation (2), the ultimate load in the single-lap fastened joint test is denoted as *P_ult_*, and *A_reduced_* is the reduced cross-section area calculated through the section with the hole. The 25 mm wide samples with one rivet are compared with the 25 mm wide samples with a 7 mm diameter hole in Figure 5 to exemplify the significance of the efficiency. It is evident in the plot that forcing the load path to go through one rivet causes a large reduction in the strength, compared with the clamped sample with a hole, and the joint with one rivet is therefore considered to have low efficiency. The stress state is represented in the diagram in Figure 5, which demonstrates that the load path goes through the rivet and into the composite via bearing on the surface of the hole. The efficiency values for all samples are provided in Table A1 and Table A2 in Appendix A, and plotted in Figure 6 and Figure 7.

*FJ = P_ult_/A_reduced_* (2)

### 3.3. Ultimate Loads and Failure Modes

**Figure 8:**
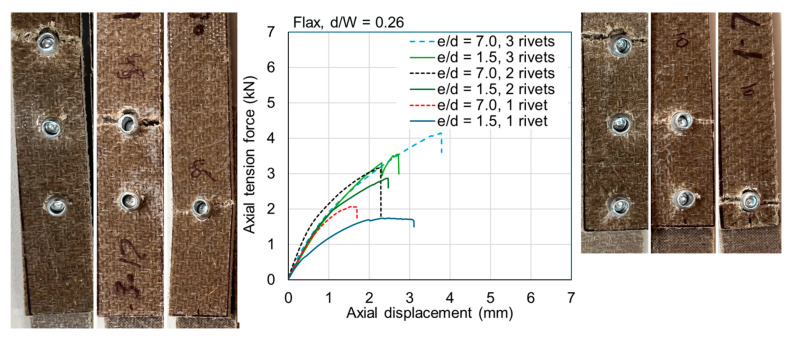
Plots of force versus axial displacement for riveted joints in narrow flax composite. Post-failure photos on the left are for edge-distance-to-diameter ratios of 7. Post-failure photos on the right are for edge-distance-to-diameter ratios of 1.5.

**Figure 9:**
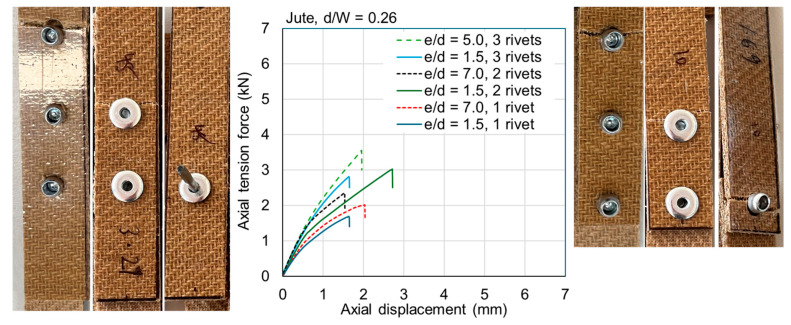
Plots of force versus axial displacement for riveted joints in narrow jute composite. Post-failure photos on the left are for edge-distance-to-diameter ratios of 7. Post-failure photos on the right are for edge-distance-to-diameter ratios of 1.5.

**Figure 10:**
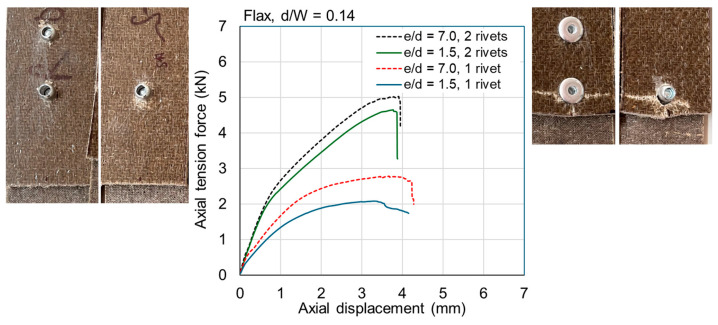
Plots of force versus axial displacement for riveted joints in wide flax composite. Post-failure photos on the left are for edge-distance-to-diameter ratios of 7. Post-failure photos on the right are for edge-distance-to-diameter ratios of 1.5.

**Figure 11:**
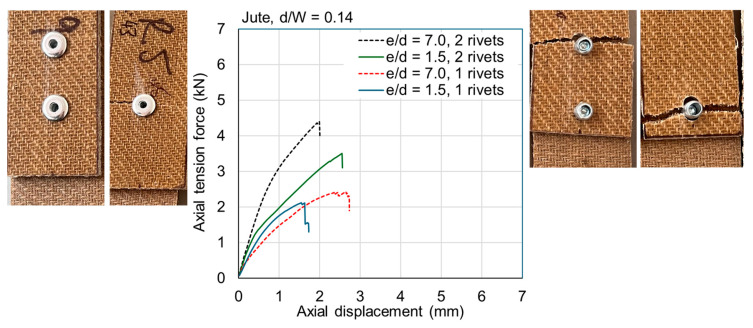
Plots of force versus axial displacement for riveted joints in wide jute composite. Post-failure photos on the left are for edge-distance-to-diameter ratios of 7. Post-failure photos on the right are for edge-distance-to-diameter ratios of 1.5.

**Figure 12:**
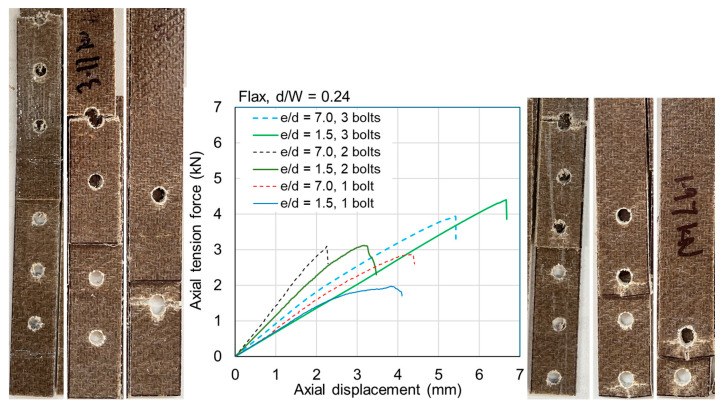
Plots of force versus axial displacement for M6 bolted joints in narrow flax composite. Post-failure photos on the left are for edge-distance-to-diameter ratios of 7. Post-failure photos on the right are for edge-distance-to-diameter ratios of 1.5.

**Figure 13:**
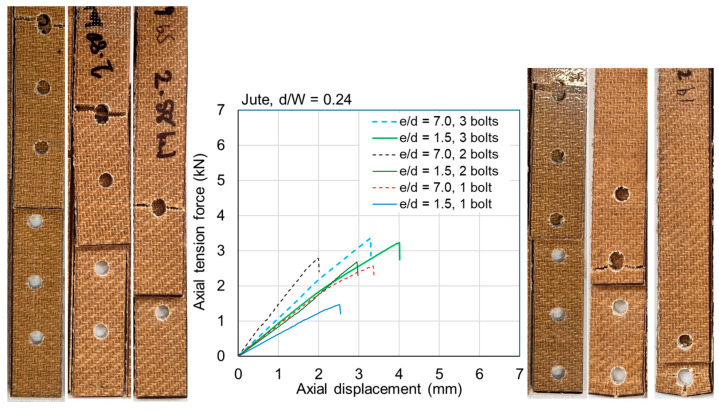
Plots of force versus axial displacement for M6 bolted joints in narrow jute composite. Post-failure photos on the left are for edge-distance-to-diameter ratios of 7. Post-failure photos on the right are for edge-distance-to-diameter ratios of 1.5.

**Figure 14:**
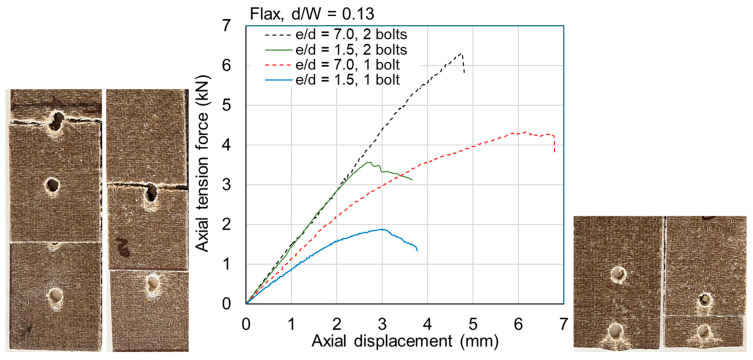
Plots of force versus axial displacement for M6 bolted joints in wide flax composite. Post-failure photos on the left are for edge-distance-to-diameter ratios of 7. Post-failure photos on the right are for edge-distance-to-diameter ratios of 1.5.

**Figure 15:**
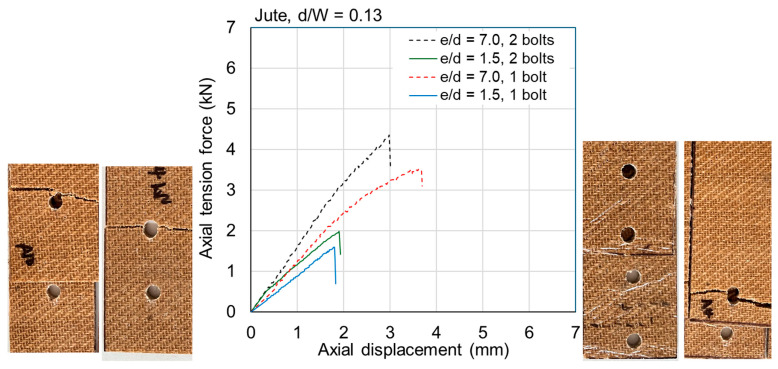
Plots of force versus axial displacement for M6 bolted joints in wide jute composite. Post-failure photos on the left are for edge-distance-to-diameter ratios of 7. Post-failure photos on the right are for edge-distance-to-diameter ratios of 1.5.

The ultimate loads are tabulated for all samples in Table A1 and Table A2 in Appendix A. Exemplar load–displacement curves with corresponding photographs of failure modes are presented in Figure 8, Figure 9, Figure 10, Figure 11, Figure 12, Figure 13, Figure 14 and Figure 15. Appendix A contains photographs of all 120 samples after testing.

**Figure 16:**
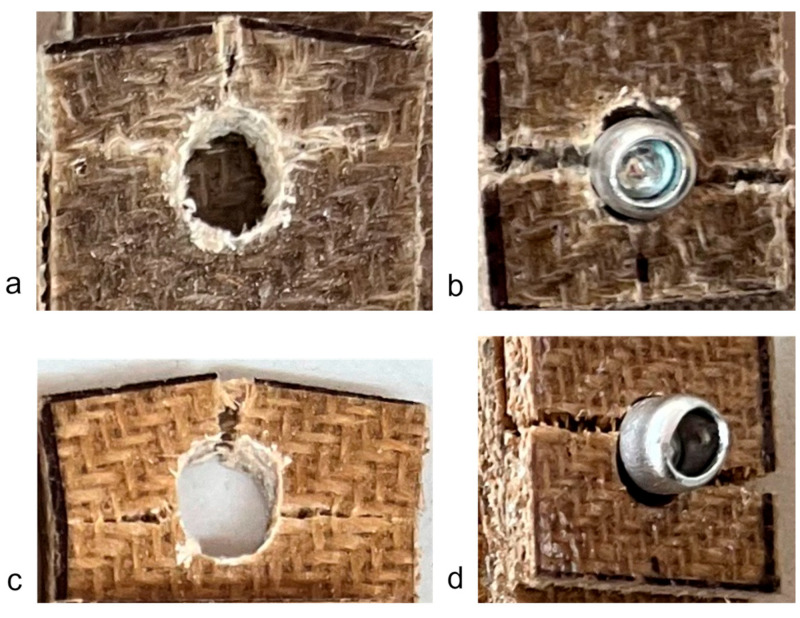
Exemplar photos of failures for *e*/*d* ratios of 1.5: (**a**) bolted joint in narrow flax composite, (**b**) riveted joint in narrow flax composite, (**c**) bolted joint in narrow jute composite, and (**d**) riveted joint in narrow jute composite.

When the fastener is located at a small distance from the edge of the sample (*e*/*d* = 1.5), the failure mode of the flax typically involved tear-out of the plate; however, some samples failed due to net section failure. This was generally true for multi-fastener joints also. For *e*/*d* = 1.5 in jute, the failure mode was typically net section failure; however, it sometimes involved plate tear-out. This difference between the materials is likely due to the fact that the jute is somewhat more brittle than the flax (Figure 4). Some close-up photographs of these failure modes are shown in Figure 16.

Fasteners located at a distance of *e*/*d* = 3.5 or greater typically failed due to net section failure, and, often, localized bearing damage was also evidenced by the elongation of the holes. Examples of such failure modes are shown in Figure 8, Figure 9, Figure 10, Figure 11, Figure 12, Figure 13, Figure 14 and Figure 15.

### 3.4. Tilting and Splaying

**Figure 17:**
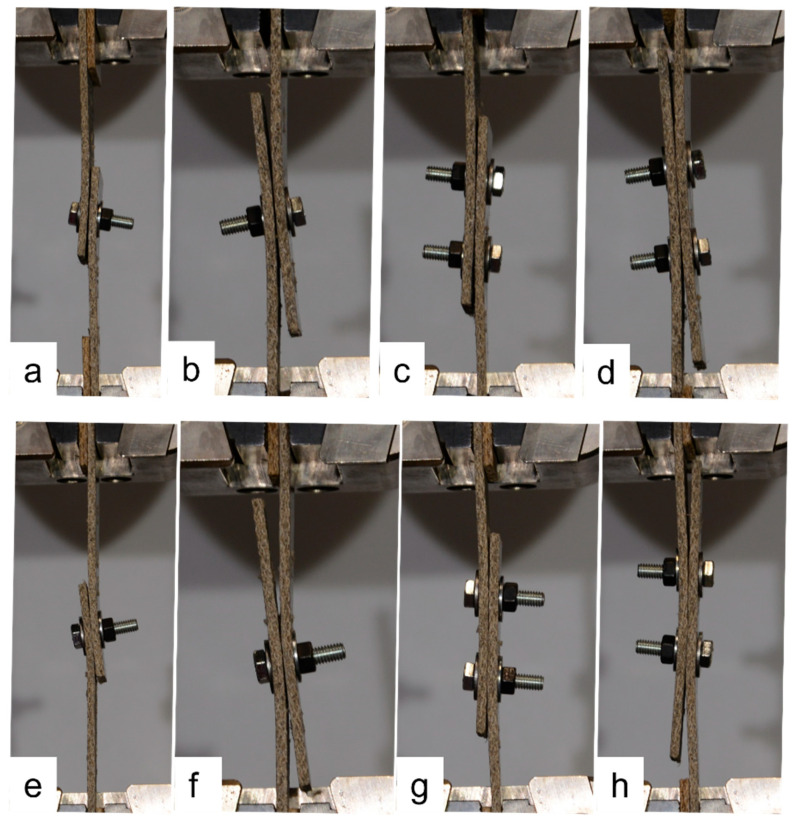
Exemplar flax composite bolted joints demonstrating varying degrees of fastener tilting and composite plate splaying: (**a**) narrow with 1 bolt and *e*/*d* = 1.5; (**b**) narrow with 1 bolt and *e*/*d* = 7; (**c**) narrow with 2 bolts and *e*/*d* = 1.5; (**d**) narrow with 2 bolts and *e*/*d* = 7; (**e**) wide with 1 bolt and *e*/*d* = 1.5; (**f**) wide with 1 bolt and *e*/*d* = 7; (**g**) wide with 2 bolts and *e*/*d* = 1.5; and (**h**) wide with 2 bolts and *e*/*d* = 7.

**Figure 18:**
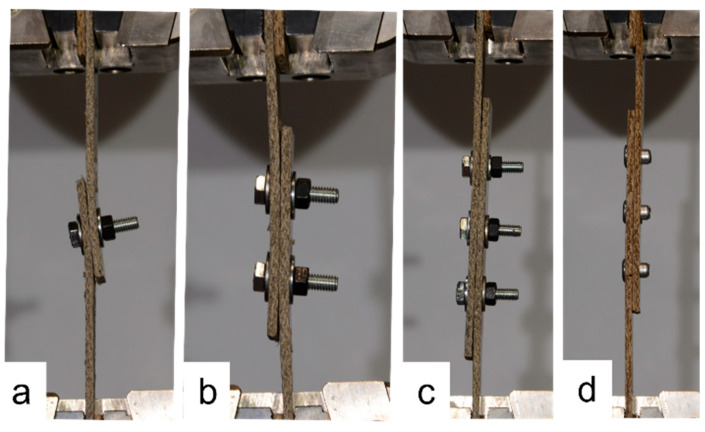
Comparison of joints with *e*/*d* = 1.5: (**a**) 1 bolt in wide flax, (**b**) 2 bolts in wide flax, (**c**) 3 bolts in narrow flax, and (**d**) 3 rivets in narrow jute.

**Figure 19:**
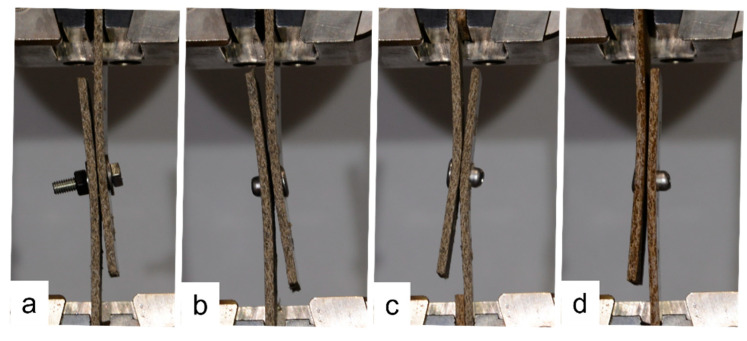
Comparison of joints with *e*/*d* = 7: (**a**) 1 bolt in narrow flax, (**b**) 1 rivet in narrow flax, (**c**) 1 rivet in wide flax, and (**d**) 1 rivet in wide jute.

The single-fastener joints displayed significant tilting, which increased as the loading continued. Fastener tilting occurred both for the narrow and wide samples, as demonstrated in Figure 17. As can be seen in Figure 17, the tilting resulted in the washer of the bolt bearing into the face of the composite plate; however, this did not occur to the extent that the bolt pulled through the plate. Similar behavior occurred in the samples with rivets. Also evident in Figure 17 is that when the *e*/*d* value is large, the composite plates splay apart as tilting occurs, due to the larger length of sample between the fastener and the plate ends. Comparing the joints with two bolts to those with one bolt in Figure 17, it is evident that tilting and splaying was reduced when two fasteners were used, and this was also true for joints with rivets. Comparing one, two, and three bolts in Figure 18, it is evident that this trend continues when a third fastener is used, in which case the tilting is negligible. Again, this was true for the joints with rivets also, as evidenced by a sample with three rivets in Figure 18. Figure 19 demonstrates the similarities between one bolt in a narrow flax sample, one rivet in a narrow flax sample, one rivet in a wide flax sample, and one rivet in a wide jute sample.

## 4. Discussion

### 4.1. Notch Sensitivity

The notch sensitivity (ratio of the Open-Hole Tension Strength to the standard Tension Strength) values of 0.86 for flax and 0.75 for jute indicate that the composites are more weakened by the hole than by the removal of material. It is well known that orthotropic materials such as fiber–matrix composites suffer from stress concentrations as a result of the presence of a hole (notch), and the material adjacent to the hole cannot reach the ultimate tensile stress of the material. This characteristic is not seen in metals, for example, where material isotropy allows the metal adjacent to the hole to reach the full ultimate Tensile Strength. This phenomenon has been described for synthetic composites; for example, notch sensitivity values of 53% for GFRP and 62% to 69% for CFRP were reported in [4,5], and values of 40% to 59% for GFRP and 45% to 53% for CFRP were reported in [40] (while one outlying CFRP laminate reached 85% in [40]). The notch sensitivity values in the present study need to be converted to compare with these values, since the studies in [4,5,40] calculated the OHT strength using the full width (whereas, in the present study, the reduced width is used, as per Equation (1)). When converted, the present values for flax and jute were 62% and 54%, respectively, which compare well with the synthetic fiber values. It is concluded therefore that the natural fiber composites in the present study had similar notch sensitivity to synthetic fiber composites reported in the literature.

### 4.2. Efficiency and the Number of Fasteners

Considering a single fastener, the efficiency of the joints was relatively low. For the following comparison, the efficiency values related to small *e*/*d* values (1.5 and 2.5) were excluded, since based on the present study and studies from the literature, a designer would seek to avoid locating a single fastener close to an edge to prevent tear-out. Table A1 and Table A2 in Appendix A and Figure 6 and Figure 7 indicate riveted joint efficiencies of 0.30–0.33 for wide flax, 0.34–0.35 for wide jute, 0.52–0.55 for narrow flax, and 0.58–0.64 for narrow jute. For bolted joints, efficiencies of 0.49–0.53 for wide flax, 0.49–0.58 for wide jute, 0.66–0.75 for narrow flax, and 0.79–0.83 for narrow jute are indicated. These data indicate that for joints with a single fastener, the composite was stronger and more efficient when loaded by a bolt compared with a rivet. This may result from damage associated with installing the rivet, the rivet clamping force, or different tilting mechanisms resulting from a different diameter washer. It is also evident that the joints with higher *d*/*W* ratios were more efficient, which was also noted by studies on synthetic composite joints, as discussed in [4].

The generally low efficiencies of single-fastener joints may be attributed to stress concentrations around the hole, while the contact force of the fastener leads to other damage mechanisms, such as matrix failure and hole elongation (and possible local fiber–matrix splitting and delamination). The eccentricity of the single-lap joint leads to fastener tilting and composite splaying. The tilting and splaying lead to additional stresses in the composite due to bending. It has been noted by several authors that in synthetic composites, the single-lap fastened joint with a single fastener results in the confluence of several different failure mechanisms [4,5].

Previous studies [4,5,6,7,8] have elucidated that the above noted-mechanisms (and other failure mechanisms relevant to composites) seen in single-fastener joints are largely alleviated in multi-fastener joints, due to a transition to predominantly net section failure. The benefits to the composite performance of using more fasteners (located along the line of action of the force) are twofold: the load carried by an individual fastener is reduced, resulting in less tilting and splaying; and lower bearing stress on the composite at the fastener locations, resulting in less matrix degradation around the hole. These benefits are clearly demonstrated in the efficiency plots in Figure 6 and Figure 7, where, in most cases, the strength and efficiency of the joints increased with the number of fasteners. Narrow samples with three fasteners generally reached the OHT strength, indicating approximately 100% efficiency. While the bolted joints were more efficient than the riveted joints in single-fastener joints, both bolted and riveted joints reached the OHT with three fasteners, indicating their performance is similar in multi-fastener joints. In [4], synthetic fiber composite multi-bolt joints were shown to reach the OHT strength with three bolts (narrow samples) or four bolts (wide samples). It is also clear in Figure 8, Figure 9, Figure 10, Figure 11, Figure 12, Figure 13, Figure 14 and Figure 15 that the stiffness generally increases with increasing number of bolts also, a result noted by other authors with synthetic fiber composites [4,5]. It is concluded, therefore, that the natural fiber composites in the present study had similar efficiency mechanisms to synthetic fiber composites reported in the literature.

### 4.3. Effect of the Sample Width

The efficiency values in Table A1 and Table A2 in Appendix A and Figure 6 and Figure 7 indicate that the narrow samples have notably higher efficiencies; that is, joint efficiency increases with the increasing *d*/*W* ratio. This has been noted by other authors with synthetic fiber composites [4] and attributed to the transition of the failure mode from bearing to net section fracture when the *d*/*W* ratio increases. While failure modes for samples with sufficient edge distance (greater than 2.5 times the fastener diameter) were typically net section failures, hole elongation and matrix damage around the hole was generally higher in the wide samples. It is concluded, therefore, that the natural fiber composites in the present study had similar joint efficiency increases with increasing *d*/*W* ratio to synthetic fiber composites reported in the literature.

### 4.4. Effect of the Edge Distance

The location of a single fastener with respect to the edge of the composite sample had an important effect on the failure mode and joint efficiency. As shown in Figure 8, Figure 9, Figure 10, Figure 11, Figure 12, Figure 13, Figure 14 and Figure 15, and in Figure 16, single fasteners near the edge often fail in a mixed bearing and tear-out mode, reducing the joint efficiency by up to approximately one-half. Interestingly, the effect is notably more pronounced in the bolted joints compared with the riveted joints. This may be related to the clamping force resulting from the insertion of the rivet.

The effect of the edge distance reduced notably in the multi-fastener joints, where two rivets had only a small reduction in efficiency, while two bolts had a large efficiency reduction in the wide samples only. Meanwhile, three fastener joints had negligible efficiency reduction due to the edge distance. The edge distance also affected the occurrence of tilting and splaying.

### 4.5. Effect of Tilting and Splaying

It is shown in Figure 17, Figure 18 and Figure 19 that single-fastener joints displayed significant tilting (fastener rotation), which increased as the loading continued. Fastener tilting and composite plate splaying became increasingly pronounced with an increase in edge distance in single-fastener joints. When the *e*/*d* value becomes large, the composite plates splay apart as tilting occurs, due to the larger length of sample between the fastener and the ends of the samples. This may result in a reduction in efficiency, since the splaying of the composite creates additional bending stresses at the fastener location. In many samples in Figure 6 and Figure 7, it may be seen that the efficiency decreases slightly at the highest *e*/*d* values. However, this effect became less pronounced in multi-fastener joints, where tilting was notably reduced due to the presence of multiple fasteners.

Studies on synthetic fiber composite single-fastener, single-lap joints have also demonstrated tilting and noted that it is detrimental to the strength as a result of the secondary bending stresses [4]. Olmedo [41] developed a model for single-fastener, single-lap joints in synthetic composites that includes a predictive method to consider the effect of secondary bending as a function of geometrical parameters, material elastic properties, stacking sequence, and load path eccentricity. In [4,5,6,7,8], it was also found that tilting was almost absent in multi-fastener joints. Tilting and secondary bending were also noted in basalt fiber composites [35].

### 4.6. Comparisons of M6 and M4 Bolted Joints

The efficiencies of the one and two fastener joints with M4 bolts were 0.31–0.32 for wide flax and 0.31–0.35 for wide jute (one bolt); and 0.53–0.55 for wide flax and 0.50–0.54 for wide jute (two bolts). These are notably lower than the efficiencies for the single M6 bolts (efficiencies of 0.49–0.53 for wide flax, and 0.49–0.58 for wide jute) and for two M6 bolts (efficiencies of 0.75–0.80 for wide flax, and 0.52–0.65 for wide jute). This indicates that for the same width of sample, lower *d*/*W* values result in lower joint efficiencies, and such outcomes may result from the fact that the load is bearing on a smaller area with the smaller bolts; therefore, the bearing stress on the composite is higher, leading to greater composite damage (such as matrix failure, hole elongation, local fiber–matrix splitting, and delamination).

### 4.7. Comparisons of Bolted and Riveted Joints

Several comparisons of bolted and riveted joints were highlighted in the previous paragraphs. In summary, in single-fastener joints, bolted joints were generally more efficient than riveted joints; when a single fastener was located close to the edge, bolted joints were less efficient than riveted joints; and, in multi-fastener joints, both bolted and riveted joints reached full efficiency (the OHT strength). Studies of single-lap, single-fastener joints in basalt fiber composites [35] found that higher clamping forces lead to improved strength, which is in agreement with the present study only for the single fasteners located near the edge (where the rivet represents the fastener with the higher clamping force).

### 4.8. Comparisons of the Present Study and Other Natural Fiber Composite Joint Studies

Kaushik [36] studied woven bidirectional flax–PP composite single- and multi-fastener joints. Joints with two bolts had 48.4% higher tensile failure load as compared to one bolt. When the diameter of the bolt was increased, the strength was 9% higher than the joints with smaller-diameter bolts, for both single- and double-bolted joints. These data compare well with the results in the present study, as did the failure modes of net section tension failure.

Sajid et al. [35] studied quasi-isotropic basalt fiber–epoxy composite single-fastener joints. Tilting and secondary bending were noted to occur in conjunction with the primarily bearing type of failures. It was noted that increasing the bolt torque increased the strength of the joints. These results are somewhat different from those of the present, notably the absence of net section tension failures in [35], and the difference in results is likely a result of differences in the composites, and the use of large washers and high torques in [35].

Choudhury et al. [34] studied bamboo fiber–PLA composite single-fastener joints. Joints in tension demonstrated an increase in strength with increases in *w*/*d*, *e*/*d*, and bolt fastening torque. Koppad et al. [42] studied natural fiber–epoxy composites fabricated with alternating layers of hemp and jute woven fabrics, joined with one or two M6 bolts. The strength of the multi-bolted joint was substantially greater than the single-bolt joint, and the failure modes were net section tension failure. These studies are generally in agreement with the results in the present study, despite consisting of different types of natural fibers.

### 4.9. Comparisons of Joints of Natural and Synthetic Fiber Composites

Several comparisons of natural and synthetic fiber composite joints were highlighted in the previous paragraphs. In summary, the natural fiber composites in the present study had similar notch sensitivity to synthetic fiber composites, similar efficiency values to synthetic fiber composites, and similar joint efficiency increases with increasing *d*/*W* ratio. The two materials also demonstrated similar failure modes, including net section failure, local bearing matrix failure, hole elongation, tilting, and splaying. It is interesting to note these similarities, despite natural and synthetic fiber composites having fundamentally different material tension characteristics. Natural fiber composites exhibit non-linearity in their tension stress–strain response and relatively higher ductility, whereas synthetic fiber composites are linear-elastic materials with lower ductility. The ultimate Tension Strengths are also substantially different, with the natural fiber composites in the present study having ultimate TSs from 69 to 73 MPa, while glass and carbon fiber composites typically have much higher values (for example, the values in [4] were 300 MPa to 500 MPa). Nonetheless, it is the conclusion of the present study that natural and synthetic fiber composites have quite similar single- and multi-fastener joint characteristics, notwithstanding that synthetic composites are substantially stronger.

## 5. Conclusions

The aim of the present study was to demonstrate the optimum strength of multi-fastener joints in natural fiber composite structural systems. To this end, the following design recommendations are proposed (for joints in which the failure is in the composite and not in the fasteners), based on the results of the 120 experiments:

1. In single-fastener joints, the fastener should be located a distance of more than 2.5 times the fastener diameter from the free edge to reduce tear-out;
2. In single-fastener joints, the fastener should be located a distance of less than seven times the fastener diameter from the free edge to reduce tilting and splaying;
3. In single-fastener joints that follow Recommendations 1 and 2, bolts are more efficient fasteners than rivets;
4. In single-fastener joints, joint efficiency increases with the increasing *d*/*W* ratio, and this ratio can be increased by either increasing the bolt diameter or decreasing the effective width (the width of the joined part that carries the bolt load);
5. Multi-fastener joints are more efficient than single-fastener joints;
6. Adding more fasteners (located along the line of action of the force), results in Recommendations 1 to 4 becoming less important, as the effects of edge distance, tilting and splaying, *d*/*W* ratio, and fastener type become less important to the joint efficiency;
7. Adding more fasteners (located along the line of action of the force) improves joint efficiency until the OHT strength is reached, which acts as an upper bound to the joint strength;
8. The OHT may be used as the design strength for multi-fastener joints with a sufficient number of fasteners (in the present study, three fasteners for a *d*/*W* ratio of 0.24).

## Figures and Tables

**Figure A1:**
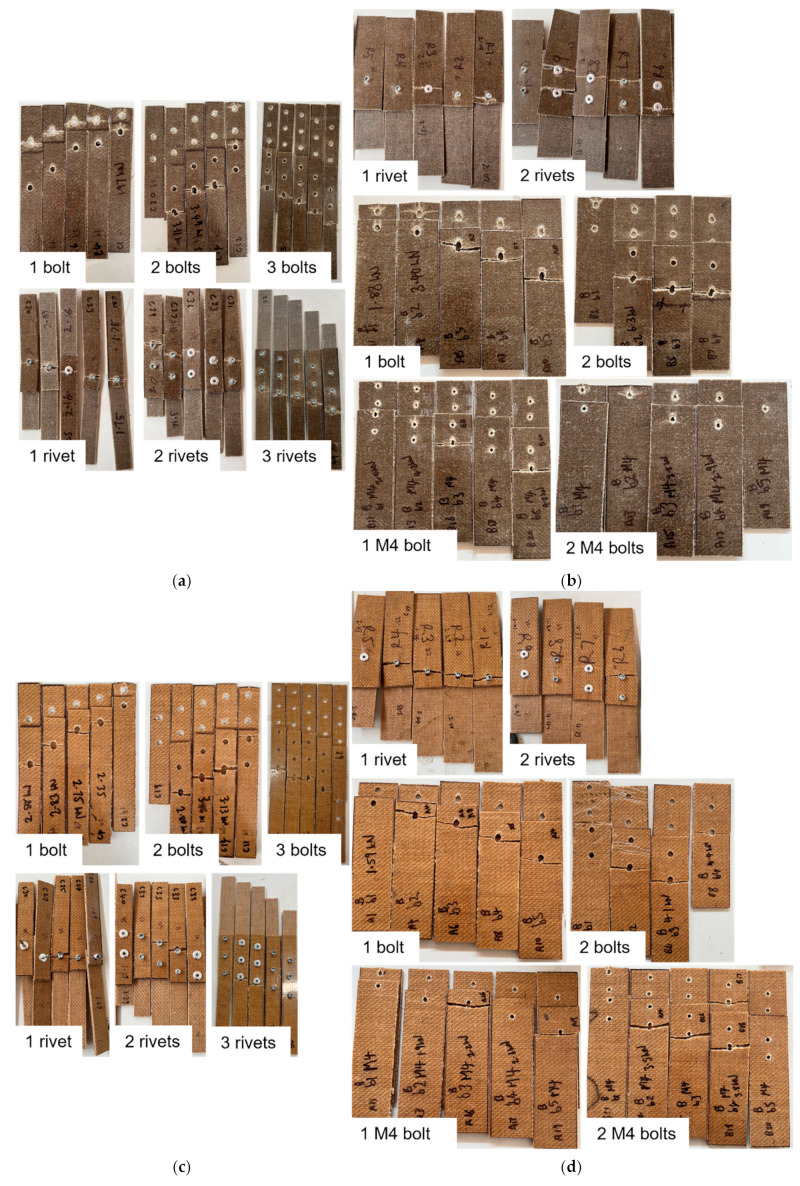
Test specimens following testing: (**a**) flax width 25 mm, (**b**) flax width 45 mm, (**c**) jute width 25 mm, and (**d**) jute width 45 mm (unless specified otherwise, bolts were M6).

## Data Availability

The original contributions presented in this study are included in the article. Further inquiries can be directed to the corresponding author.

## Appendix A

**Table A1:** Geometry and test results for wide samples (*W* = 45 mm; thickness = 3.5 mm).

			Nominal	Nominal	Jute	Flax	Jute	Jute	Flax	Flax	Jute	Flax
Fastener Type	Fastener Diameter	Number of Fasteners	Edge Distance /Diameter	Diameter /Width	Ultimate Force	Ultimate Force	Width	Ultimate Stress	Width	Ultimate Stress	Efficiency	Efficiency
	(mm)		(*e*/*d*)	(*d*/*W*)	(kN)	(kN)	(mm)	(MPa)	(mm)	(MPa)		
Rivet	6.4	1	1.5	0.14	2.1	2.1	45.5	15.8	45.5	15.5	0.30	0.25
Rivet	6.4	1	2.5	0.14	2.3	2.6	46.4	16.6	45.9	19.3	0.32	0.31
Rivet	6.4	1	3.5	0.14	2.4	2.5	45.5	18.1	45.0	18.9	0.35	0.30
Rivet	6.4	1	5	0.14	2.4	2.7	45.0	18.3	46.8	19.6	0.35	0.31
Rivet	6.4	1	7	0.14	2.4	2.8	45.5	18.1	45.5	20.7	0.35	0.33
Rivet	6.4	2	1.5	0.14	3.5	4.7	45.5	26.0	46.4	33.8	0.50	0.54
Rivet	6.4	2	2.5	0.14	4.3	5.2	46.4	31.5	45.0	39.3	0.61	0.63
Rivet	6.4	2	3.5	0.14	4.4	4.9	46.4	32.0	45.0	36.9	0.62	0.59
Rivet	6.4	2	5	0.14	4.0	5.1	46.8	28.8	45.0	38.6	0.56	0.62
Rivet	6.4	2	7	0.14	4.0	5.0	46.8	28.4	45.0	37.8	0.55	0.61
Bolt	6	1	1.5	0.13	1.6	1.9	44.1	12.3	45.9	13.8	0.24	0.22
Bolt	6	1	2.5	0.13	3.0	3.4	44.6	22.5	44.6	26.0	0.43	0.42
Bolt	6	1	3.5	0.13	3.9	4.3	44.1	30.1	46.8	30.7	0.58	0.49
Bolt	6	1	5	0.13	3.7	4.5	45.5	27.3	45.9	32.9	0.53	0.53
Bolt	6	1	7	0.13	3.5	4.3	46.8	25.2	46.4	31.5	0.49	0.50
Bolt	6	2	1.5	0.13	2.0	3.6	45.0	14.9	45.9	26.2	0.29	0.42
Bolt	6	2	2.5	0.13	4.8	6.3	46.8	34.5	44.1	48.8	0.66	0.78
Bolt	6	2	3.5	0.13	4.1	6.2	44.1	31.9	45.0	46.5	0.61	0.75
Bolt	6	2	5	0.13	4.4	6.3	44.1	33.7	45.5	46.8	0.65	0.75
Bolt	6	2	7	0.13	3.5	6.6	44.4	27.0	44.8	49.9	0.52	0.80
Bolt	4	1	1.5	0.09	1.2	1.3	46.4	8.2	45.9	9.0	0.16	0.14
Bolt	4	1	2.5	0.09	1.9	2.4	46.8	13.3	44.1	17.2	0.26	0.28
Bolt	4	1	3.5	0.09	2.2	2.8	44.1	16.0	45.0	19.8	0.31	0.32
Bolt	4	1	5	0.09	2.6	2.9	45.5	18.1	45.9	20.2	0.35	0.32
Bolt	4	1	7	0.09	2.3	2.8	45.0	16.5	45.9	19.6	0.32	0.31
Bolt	4	2	1.5	0.09	1.7	2.0	44.1	12.5	45.9	14.1	0.24	0.23
Bolt	4	2	2.5	0.09	3.5	4.3	44.6	25.1	46.4	29.4	0.48	0.47
Bolt	4	2	3.5	0.09	3.6	4.6	45.0	25.8	44.6	33.0	0.50	0.53
Bolt	4	2	5	0.09	4.0	4.8	44.6	28.5	45.5	33.9	0.55	0.54
Bolt	4	2	7	0.09	3.8	4.8	44.1	27.9	45.5	34.2	0.54	0.55

**Table A2:** Geometry and test results for narrow samples (*W* = 25 mm; thickness = 3.5 mm).

			Nominal	Nominal	Jute	Flax	Jute	Jute	Flax	Flax	Jute	Flax
Fastener Type	Fastener Diameter	Number of Fasteners	Edge Distance /Diameter	Diameter /Width	Ultimate Force	Ultimate Force	Width	Ultimate Stress	Width	Ultimate Stress	Efficiency	Efficiency
	(mm)		(*e*/*d*)	(*d*/*W*)	(kN)	(kN)	(mm)	(MPa)	(mm)	(MPa)		
Rivet	6.4	1	1.5	0.26	1.7	1.7	25.5	26.1	25.8	26.6	0.50	0.43
Rivet	6.4	1	2.5	0.26	1.8	2.1	25.8	27.2	25.5	32.9	0.52	0.53
Rivet	6.4	1	3.5	0.26	2.0	2.2	25.8	29.9	25.5	33.3	0.58	0.53
Rivet	6.4	1	5	0.26	2.0	2.1	24.5	33.3	24.5	34.2	0.64	0.55
Rivet	6.4	1	7	0.26	2.0	2.1	26.0	30.2	25.3	32.5	0.58	0.52
Rivet	6.4	2	1.5	0.26	3.0	2.9	24.8	48.7	25.8	44.3	0.94	0.71
Rivet	6.4	2	2.5	0.26	3.0	2.9	25.0	47.4	24.5	46.9	0.91	0.75
Rivet	6.4	2	3.5	0.26	2.9	3.4	24.5	47.5	26.0	50.8	0.91	0.81
Rivet	6.4	2	5	0.26	2.9	3.2	25.0	46.0	25.8	48.2	0.89	0.77
Rivet	6.4	2	7	0.26	3.3	3.0	25.3	51.5	25.3	47.0	0.99	0.75
Rivet	6.4	3	1.5	0.26	2.8	4.1	25.3	43.9	25.0	65.8	0.95	1.05
Rivet	6.4	3	2.5	0.26	3.8	4.1	25.0	60.0	25.3	64.6	1.16	1.03
Rivet	6.4	3	3.5	0.26	3.6	4.0	25.0	57.2	26.0	60.5	1.10	0.97
Rivet	6.4	3	5	0.26	3.6	4.2	25.8	54.1	25.3	65.4	1.04	1.05
Rivet	6.4	3	7	0.26	3.3	3.6	26.0	50.0	25.8	54.2	0.96	0.87
Bolt	6	1	1.5	0.24	1.5	2.0	24.8	23.6	25.8	30.0	0.45	0.48
Bolt	6	1	2.5	0.24	2.4	2.7	26.0	35.4	24.5	43.6	0.68	0.70
Bolt	6	1	3.5	0.24	2.8	2.7	25.5	42.5	25.5	41.2	0.82	0.66
Bolt	6	1	5	0.24	2.8	3.0	25.8	43.1	25.5	46.6	0.83	0.75
Bolt	6	1	7	0.24	2.6	2.9	25.0	40.9	25.0	45.5	0.79	0.73
Bolt	6	2	1.5	0.24	2.7	3.1	24.8	43.3	24.5	51.0	0.83	0.82
Bolt	6	2	2.5	0.24	3.1	3.3	25.3	49.0	25.5	51.3	0.94	0.82
Bolt	6	2	3.5	0.24	3.2	3.5	26.0	47.6	24.5	57.1	0.92	0.91
Bolt	6	2	5	0.24	2.8	3.1	25.0	44.5	25.3	48.7	0.86	0.78
Bolt	6	2	7	0.24	2.5	3.2	25.0	39.4	24.5	53.1	0.76	0.85
Bolt	6	3	1.5	0.24	3.2	4.4	25.0	51.4	25.8	67.0	0.99	1.05
Bolt	6	3	2.5	0.24	3.4	4.2	25.5	51.8	25.5	64.5	1.00	1.03
Bolt	6	3	3.5	0.24	3.5	3.9	25.0	55.6	24.5	64.4	1.07	1.03
Bolt	6	3	5	0.24	3.3	3.9	25.8	50.4	24.8	63.4	0.97	1.02
Bolt	6	3	7	0.24	3.4	3.9	25.5	51.9	25.8	60.0	1.00	0.96

## References

[1] Waszczak J.P., Cruse T.A. (1971). Failure mode and strength predictions of anisotropic bolt bearing specimens. J. Compos. Mater..

[2] Stockdale J.H., Matthews F.L. (1976). The effect of clamping pressure on bolt bearing loads in glass fibre reinforced plastics. Composites.

[3] Duthinh D. (2000). Connections of Fiber-Reinforced Polymer (FRP) Structural Members: A Review of the State of the Art.

[4] Gamdani F., Boukhili R., Vadean A. (2015). Tensile strength of open-hole, pin-loaded and multi-bolted single-lap joints in woven composite plates. Mater. Des..

[5] Gamdani F., Boukhili R., Vadean A. (2019). Tensile behavior of hybrid multi-bolted/bonded joints in composite laminates. Int. J. Adhes. Adhes..

[6] Yun J.H., Choi J.H., Kweon J.H. (2014). A study on the strength improvement of the multi bolted joint. Compos. Struct..

[7] Feo L., Marra G., Mosallam A.S. (2012). Stress analysis of multi-bolted joints for FRP pultruded composite structures. Compos. Struct..

[8] Zhang J., Liu F., Zhao L., Fei B. (2014). A novel characteristic curve for failure prediction of multi-bolt composite joints. Compos. Struct..

[9] Summerscales J., Dissanayake N.P.J., Virk A.S., Hall W. (2010). A review of bast fibers and their composites. Part 1—Fibers as reinforcements. Compos. Part A.

[10] Summerscales J., Dissanayake N.P.J., Virk A.S., Hall W. (2010). A review of bast fibers and their composites. Part 2—Composites. Compos. Part A.

[11] Ku H., Wang H., Pattarachaiyakoop N., Trada M. (2011). A review on the tensile properties of natural fiber reinforced polymer composites. Compos. Part B Eng..

[12] Yan L.B., Chouw N., Jayaraman K. (2014). Flax fibe and its composites—A review. Compos. Part B.

[13] Pickering K.L., Efendy M.G.A., Le T.M. (2016). A review of recent developments in natural fiber composites and their mechanical performance. Compos. Part A.

[14] Faruk O., Bledzki A.K., Fink H.-P., Sain M. (2012). Biocomposites reinforced with natural fibers: 2000–Prog. Polym. Sci..

[15] Dicker M.P.M., Duckworth P.F., Baker A.B., François G., Hazzard M.K., Weaver P.M. (2014). Green composites: A review of material attributes and complementary applications. Compos. Part A Appl. Sci. Manuf..

[16] Weclawski B.T., Fan M., Hui D. (2014). Compressive behaviour of natural fiber composite. Compos. Part B.

[17] Costa F.H.M.M., D’Almeida J.R.M. (1999). Effect of Water Absorption on the Mechanical Properties of Sisal and Jute Fiber Composites. Polym. Technol. Eng..

[18] Hargitai H., Rácz I., Anandjiwala R.D. (2008). Development of HEMP Fiber Reinforced Polypropylene Composites. J. Thermoplast. Compos. Mater..

[19] Oksman K. (2001). High quality flax fiber composites manufactured by the resin transfer moulding process. J. Reinf. Plast. Comp..

[20] Da Silva H.S.P., Júnior H.L.O., Júnior J.H.A., Zattera A.J., Amico S.C. (2013). Mechanical behavior and correlation between dynamic fragility and dynamic mechanical properties of curaua fiber composites. Polym. Compos..

[21] Da Silva L.V., Júnior J.H.A., Angrizani C.C., Amico S.C. (2012). Short beam strength of curaua, sisal, glass and hybrid composites. J. Reinf. Plast. Compos..

[22] Bambach M.R. (2017). Compression strength of natural fiber composite plates and sections of flax, jute and hemp. Thin Walled Struct..

[23] Bambach M.R. (2018). Geometric optimisation and compression design of natural fiber composite structural channel sections. Compos. Struct..

[24] Bambach M. (2020). Durability of natural fibre epoxy composite structural columns: High cycle compression fatigue and moisture ingress. Compos. Part C Open Access.

[25] Shah D.U., Schubel P.J., Clifford M.J. (2013). Can flax replace E-glass in structural composites? A small wind turbine blade case study. Compos. Part B Eng..

[26] Yan L.B., Chouw N. (2013). Crashworthiness characteristics of flax fiber reinforced epoxy tubes for energy absorption application. Mat. Des..

[27] Yan L.B., Chouw N. (2013). Behaviour and analytical modelling of natural flax fiber reinforced polymer tube encased coir fiber reinforced concrete composite column. J. Comp. Mater..

[28] Mak K., Fam A., MacDougall C. (2015). Flexural Behavior of Sandwich Panels with Bio-FRP Skins Made of Flax Fibers and Epoxidized Pine-Oil Resin. J. Compos. Constr..

[29] Le Duigou A., Deux J.-M., Davies P., Baley C. (2010). PLLA/Flax Mat/Balsa Bio-Sandwich Manufacture and Mechanical Properties. Appl. Compos. Mater..

[30] Uddin N., Kalyankar R. (2011). Manufacturing and Structural Feasibility of Natural Fiber Reinforced Polymeric Structural Insulated Panels for Panelized Construction. Int. J. Polym. Sci..

[31] Lansiaux H., Soulat D., Boussu F., Labanieh A.R. (2020). Development and Multiscale Characterization of 3D Warp Interlock Flax Fabrics with Different Woven Architectures for Composite Applications. Fibers.

[32] Wahab N., Srinophakun P., Hussain Q., Chaimahawan P. (2019). Performance of Concrete Confined with a Jute–Polyester Hybrid Fiber Reinforced Polymer Composite: A Novel Strengthening Technique. Fibers.

[33] Manaia J.P., Manaia A.T., Rodriges L. (2019). Industrial Hemp Fibers: An Overview. Fibers.

[34] Choudhury M.R., Debnath K. (2019). Experimental analysis of tensile and compressive failure load in single-lap bolted joint of green composites. Compos. Struct..

[35] Sajid Z., Karuppanan S., Shah S.Z.H. (2021). Effect of Washer Size and Tightening Torque on Bearing Performance of Basalt Fiber Composite Bolted Joints. J. Nat. Fibers.

[36] Kaushik D., Singh I. (2024). Comparative assessment of failure in single shear lap joints fabricated using various joining techniques. Eng. Fail. Anal..

[37] Malik K., Ahmad F., Gunister E. (2022). Drilling Performance of Natural Fiber Reinforced Polymer Composites: A Review. J. Nat. Fibers.

[38] (2023). Standard Test Method for Bearing Response of Polymer Matrix Composite Laminates.

[39] (2021). Part 4: Test Conditions for Isotropic and Orthotropic Fibre-Reinforced Plastic Composites.

[40] O’Higgins R.M., McCarthy M.A., McCarthy C.T. (2008). Comparison of open-hole tension characteristics of high strength glass and carbon fibre-reinforced composite materials. Compos. Sci. Technol..

[41] Olmedo A., Santiuste C., Barbero E. (2014). An analytical model for the secondary bending prediction in single-lap composite bolted-joints. Compos. Struct..

[42] Koppad P., Suryanarayana R.S., Reddy N., Sethuram D. (2024). Experimental Studies on Mechanical and Failure Behaviour of Single Lap Joints of Woven Jute-Hemp Fabric Reinforced Polymeric Composite Laminates.

